# Sedative/Tranquilizer Misuse is Associated With Alcohol and Illicit Drug Problems, Mental Health Issues, and Impulsivity and Compulsivity in University Students

**DOI:** 10.1097/ADM.0000000000000556

**Published:** 2019-08-08

**Authors:** Jon E. Grant, Katherine Lust, Samuel R. Chamberlain

**Affiliations:** Department of Psychiatry & Behavioral Neuroscience, University of Chicago, Chicago, IL (JEG); Boynton Health Service, University of Minnesota, MN (KL); Department of Psychiatry, University of Cambridge; & Cambridge and Peterborough NHS Foundation Trust (CPFT), UK (SRC).

**Keywords:** addiction, drugs, illicit, impulsivity, sedatives, tranquilizers, well-being

## Abstract

**Background:**

This study examined the prevalence of sedative/tranquilizer misuse among university students and its associations with psychosocial correlates.

**Methods:**

Nine thousand four hundred forty-nine students received a 156-item anonymous online survey, which assessed the use of prescription sedative/tranquilizer (ever or past year), alcohol and drug use, mental health issues, and impulsive and compulsive traits. Sedative/tranquilizer misuse was defined as intake of these prescription drugs by individuals who had not been prescribed them.

**Results:**

Three thousand five hundred twenty-five university students (57.7% women) responded to the survey. The prevalence of past 12-month prescription sedative/tranquilizer misuse was 2.1%, with 2.8% reporting having used more than 12 months ago. Prescription sedative/tranquilizer misuse was associated with the use of multiple other drugs (eg, alcohol, opiates each *P* < 0.001). Those who misuse sedative/tranquilizers were significantly more likely to have mental health histories (*P* < 0.001), engage in riskier sexual behavior (ie, earlier sexual acts [*P* < 0.001] and less frequent use of barrier contraception [*P* = 0.001]), report low self-esteem (*P* = 0.001), and endorse traits of impulsivity (*P* < 0.001) and compulsivity (*P* < 0.001). Effect sizes were small to medium.

**Conclusions:**

Misuse of prescription sedative/tranquilizers was reported by 2% to 3% of university students and was associated with a variety of mental health and drug use problems. Clinicians should be aware that certain mental health conditions are more likely in those who misuse sedatives. This study indicates the need for longitudinal research into the effects of chronic sedative use on brain function and mental health, especially in young people. Such research should address the extent to which impulsive traits predispose to various substance use problems, versus the direct effects of sedatives (and other substances) on mental health

Data indicate that the use of prescription sedatives and tranquilizers in the United States has increased over the last 15 years (Agarwal and Landon, 2019), and with the increase in prescriptions has come an increase in the misuse of these drugs (Ford and McCutcheon, 2012; Rigg and Ford, 2014). In fact, the National Survey on Drug Use and Health (NSDUH) estimated that among young adults ages 18 to 25 years, approximately 1.6% misused tranquilizer medication and 0.2% misused sedative medication in the past month (SAMHSA, 2017). Similarly, the National Epidemiologic Survey on Alcohol and Related Conditions (NESARC) found that 5.5% of adults had misused prescription tranquilizer or sedative medications in their lifetimes, with an estimated mean age at onset of approximately 24.2 years (Boyd et al., 2018). Overdoses of sedative medications constitutes a leading cause of drug overdose deaths in the USA (https://www.drugabuse.gov/related-topics/trends-statistics/overdose-death-rates). Although the misuse of sedatives/tranquilizers is a public health concern, the associated psychological and psychosocial consequences of sedative/tranquilizer misuse remains understudied.

Misuse of sedatives/tranquilizers is not only a problem on its own, but also frequently presents alongside other substance use issues. For example, a study of wounded veterans (n = 212) found rates of past year prescription sedative misuse was 21.7%, and that nearly all participants who misused sedatives (97.8%) also misused opioids in the past year (Kelley et al., 2018). This co-occurrence of problematic use of substances is, particularly, important given the potential fatal consequences of sedative/tranquilizer misuse. In the United States, fatal benzodiazepine overdose increased by over 400% from 1996 to 2013 (Bachhuber et al., 2016), with a nearly 300% increase because of benzodiazepine and opioid co-ingestion (Jones and McAninch, 2015), and emergency department visits increasing by over 300% from 2005 to 2011 because of benzodiazepines and opioid co-ingestion (Jones and McAninch, 2015). Furthermore, the number of opioid-related deaths is increasing at an alarming rate in the USA over time (Gomes et al., 2018).

The misuse of sedatives/tranquilizers may be particularly relevant to older adolescents and young adults. One study examining age cohorts and sedative/tranquilizer misuse found that lifetime misuse rates were the highest in adults ages 26 to 34 years (8.1%), but past year and past month tranquilizer/sedative misuse rates were the highest in young adults ages 18 to 25 years (5.8% and 1.8%, respectively; Schepis et al., 2018). There is sparse information, however, on the mental health issues of adolescents/young adults who misuse sedatives/tranquilizers. In studies of adolescents, tranquilizer/sedative misuse was associated with depression, poorer academic achievement, and problematic substance use (Schepis and Krishnan-Sarin, 2008, Hall et al., 2010). These data were from n = 723 Missouri youth in residential care for antisocial behavior; and n = 18,678 adolescents from the 2005 National Survey on Drug Use and Health, respectively. In a study of n = 729 medical students, sedative drug use was associated with lower grade point averages (Al-Sayed et al., 2014); and in n = 509 college students, multiple logistic regression found that anxiolytic/sedative use was associated with regretted sexual activity (Parks et al., 2017).

In view of the relative lack of research in this important area of public health, the current study sought to examine both the prevalence of the misuse of sedatives/tranquilizers among university students; and to examine related behaviors and mental health issues. On the basis of the previous literature, we hypothesized that the misuse of sedatives/tranquilizers would be associated with elevated rates of other substance use (including a range of illicit drug use), mental health issues, trait impulsivity with compulsivity, riskier sexual practices (earlier first sexual activity, and less use of barrier contraception), and academic impairments compared with students who do not misuse sedatives/tranquilizers. Our hypotheses in relation to self-report impulsivity and compulsivity derived from previous work implicating these 2 features in the instantiation and persistence of substance use problems more broadly (Yucel et al., 2018). According to this expert consensus, developed using the Delphi Technique, impulsivity and compulsivity are core domains implicated in addictive behaviours, but it was suggested that impulsivity is more related to symptom onset, and compulsivity is more related to chronicity (Yucel et al., 2018).

## METHODS

### Survey Design

Researchers at the Department of Psychiatry and Behavioral Neuroscience at the University of Chicago and Boynton Health Services at the University of Minnesota jointly developed the *Health and Addictive Behaviors Survey,* an online survey examining the use of alcohol, drugs, and mental health issues, in university students. The study was ethically approved by Institutional Review Board (University of Minnesota).

### Participants

Ten thousand undergraduate and graduate/professional students at a large Midwestern university were chosen randomly using a computer-generated selection with email addresses and sent an online survey during a 3-week period in the Autumn of 2016. Of the 10,000 email invitations, 9449 were successfully received by the recipients. The survey first presented students with information sheets about the study (including informing them that all information was anonymous and confidential). Students then provided consent to take part, or opted out. Subsequent questionnaires were only presented when informed consent had been provided.

Students were also informed that completing the survey would result in their email addresses (to maintain anonymity, the email addresses were not linked to questionnaire responses) being entered into a raffle wherein 10 students would be randomly chosen to receive prizes: 3 would win tablet computers, 4 would win $250 gift certificates to an online retailer, 2 would win $500 gift certificates, and there would be a single winner of a $1000 gift certificate. Participants were required to review all survey questions to be eligible for the prize drawings. Students were not required, however, to answer all questions, given the sensitive nature of some items.

Of the 9449 students who received the invitation to participate, 3525 (37.3%) completed the survey, a response rate in keeping with other health surveys (Cook et al., 2000; Baruch and Holtom, 2008; Van Horn et al., 2009). All study procedures were conducted in accordance with the Declaration of Helsinki and the University of Minnesota's Institutional Review Board approved the study.

### Assessments

The survey consisted of 156 questions and took approximately 30 minutes to complete. Sedative/tranquilizer misuse was assessed by asking participants if they had used prescription tranquilizers/sedatives (eg, benzodiazepine medications, such as alprazolam, and medications primarily indicated for insomnia, such as zolpidem) that were not prescribed in the past year or used ever in their lifetime. Participants were grouped into “current” sedative/tranquilizer misuse if they reported using any in the last 12 months, those who misused prescription sedative/tranquilizer medication previously, but not in last 12 months, were labeled as “past” prescription sedative/tranquilizer medication misuse. Those who never misused prescription sedative/tranquilizer medications constituted the third category. The term ‘misuse’ has been used to describe 2 different behaviors that are often reported in the literature, in the first case, the use of prescription medications that are not prescribed to the user, or in the second case, the use of prescription medication in a manner not intended by the prescriber (eg, using too much, using to get high). This survey asked only about the first type of misuse.

The following demographic measures were collected: sex, years in college, whether in full time education, and grade point average (GPA). In addition to asking demographic and clinical information, including information about sexual practices, the survey used measures of interest focusing on 3 domains: drug and alcohol use; mental health problems; and impulsivity/compulsivity.

#### Drug and Alcohol Use

Participants were asked if they had ever used an illicit drug (binary); and were asked about whether they had used the following in the past 12 months (each a binary response): amphetamines, cocaine, heroin, hallucinogens, marijuana or hashish, prescription opioid pain medication, or sedatives. In addition to use of drugs and alcohol, participants were screened for possible problematic use by using the *Alcohol Use Disorders Identification Test (AUDIT)* (10 questions; a score of ≥8 indicating potentially harmful alcohol use (Saunders et al., 1993); and the *Drug Abuse Screening Test (DAST-10)* (10 questions; a score of 3 indicates a positive screen for a drug use disorder; Skinner, 1982; Yudko et al., 2007).

#### Mental Health Problems

*Patient Health Questionnaire* (*PHQ-9*; a 9-items; score of ≥10 indicating depressive symptoms of moderate or higher severity; Kroenke et al., 2001); *Generalized Anxiety Disorder 7* (*GAD-7*; 7 questions; score of 10 or greater indicating clinically significant anxiety; Spitzer et al., 2006); *Primary Care PTSD Screen* (*PC-PTSD*; 4 questions; score of ≥3 indicating probable posttraumatic stress disorder, PTSD; Prins et al., 2003); *Adult ADHD Self-Report Scale (ASRS-v1.1) Part A* (6 questions screening for attention-deficit/hyperactivity disorder [ADHD]; Kessler et al., 2005, 2007); *Minnesota Impulsive Disorders Interview (MIDI)* (screen for specific impulse control disorders, including compulsive sexual behavior, binge eating disorder, and gambling disorder; Grant, 2008, Chamberlain and Grant, 2018b); and the *Rosenberg Self-Esteem Scale* (*RSES*; 10-items; score <15 indicating low self-esteem; Rosenberg, 1965).

#### Impulsivity/Compulsivity

Impulsivity refers to a tendency towards inappropriate, premature, unduly hasty acts (Evenden, 1999); whereas compulsivity refers to a tendency towards repetitive habitual actions (Chamberlain et al., 2018a). *Barratt Impulsiveness Scale, Version 11 (BIS-11)* (30 items; 3 dimensions of impulsivity—attentional, motor, and nonplanning; Patton et al., 1995; Stanford et al., 2016); and the *Cambridge-Chicago Compulsivity Trait Scale (CHI-T)* (15 questions; compulsive traits; Chamberlain and Grant, 2018a).

### Data Analysis

Participants were grouped a priori into current, past or nonmisusers per the definitions provided above under “participants.” Categorical variables were assessed using Pearson's chi-squared test. Continuous variables were assessed using Analysis of Variance (ANOVA). Effect size was determined using Cramer's V or Cohen's D as appropriate. Our primary aim was to show how the groups actually presented, rather than to statistically control for potential covariates, as the former approach is intuitive to clinicians and more likely to be relevant practically both to individuals who misuse sedative/tranquilizers and to healthcare professionals seeing such people. SPSS was used for all statistical analyses (version 24; IBM Corp). Raw *P* values were reported but findings were only deemed statistically significant if they withstood Bonferroni correction at *P* < 0.05 2-tailed for the number of measures within a given category of interest (ie, per table of results).

Missing data were missing completely at random (MCAR) and the analysis was conducted using listwise deletion. By far the most common approach to the missing data is to simply omit those cases with the missing data and analyze the remaining data. This approach is known as the complete case (or available case) analysis or listwise deletion. Listwise deletion is the most frequently used method in handling missing data. Although this may introduce bias in the estimation of the parameters, if the assumption of MCAR is satisfied, a listwise deletion is known to produce unbiased estimates and conservative results. Also, because this was a large sample, where power was not an issue, the assumption of MCAR was satisfied and listwise deletion was thus appropriate.

## RESULTS

Of the 3525 university students (57.7% women) the overall prevalence of past 12-month sedative/tranquilizer misuse was 2.1%, whereas an additional 2.8% reported lifetime use but not in the past year. Of those surveyed, 73 had used sedative/tranquilizers within the past 12 months, 94 had used sedative/tranquilizers more than 12 months ago, and 3358 had never used sedative/tranquilizers. Demographic characteristics of the groups are presented in Table 1. It can be seen that those who reported misuse (ever misuse/past year misuse) of sedative/tranquilizers had significantly lower GPAs (ie, lower educational achievement scores from examinations).

**TABLE 1:** Demographics of University Students Based on Nonmedical Use of Sedatives/Tranquilizers

Variable	Students Who Currently Misuse Prescription Sedative/Tranquilizers (n = 73)	Students Who Have Misused Prescription Sedative/Tranquilizers in the Past (n = 94)	Students Who Have Never misused prescription Sedative/Tranquilizers (n = 3358)	Statistic Likelihood Ratio	*P* Value	Effect SizeCramer V
Sex, female, n (%)	46 (63.9)	49 (55.1)	1940 (60.9)	LR = 4.566; df = 6	0.601	0.030
Year in college, n (%)						
Undergraduate	52 (71.2)	63 (67.0)	2204 (65.6)	LR = 2.159; df = 4	0.707	0.011
Graduate	21 (28.8)	30 (31.9)	1135 (33.8)			
Non-degree	0 (0.0)	1 (1.1)	19 (0.6)			
Race/ethnicity, Caucasian	56 (77.8)	71 (79.8)	2402 (75.4)	LR = 1.124; df = 2	0.570	0.018
Full time student, n (%)	68 (93.2)	75 (79.8)	3103 (92.5)	LR = 14.892; df = 2	0.001 ^*^	0.076
Grade Point Average, GPA						
Less than 3.00	11 (15.1)	19 (20.2)	331 (10.0)	LR = 10.008; df = 2	0.007 ^*^	0.059
3.00 or higher	62 (84.9)	75 (79.8)	2986 (90.0)			

^*^P < 0.05, Bonferroni-corrected. Df, degrees of freedom; LR, likelihood ratio.

Sedative/tranquilizer misuse was significantly associated with higher levels of problematic alcohol and illicit substance use (AUDIT and DAST-10), with approximately two-thirds of those currently misusing sedative/tranquilizers meeting the threshold for a current alcohol or drug addiction (though the drug addiction would also include some of these young adults who may be addicted to sedatives/tranquilizers). In addition, sedative/tranquilizer misuse was significantly associated with a greater likelihood of using numerous substances, even if not problematic (see Table 2).

**TABLE 2:** Alcohol, Tobacco, and Illicit Drug Use in Students Based on Nonmedical Use of Prescription Sedative/Tranquilizers

Variable	Students Who Currently Misuse Prescription Sedative/Tranquilizer (n = 73)	Students Who Have Misused Prescription Sedative/Tranquilizer in the Past (n = 94)	Students Who Have Never Misused Prescription Sedative/Tranquilizer (n = 3358)	StatisticLikelihood Ratio	*P* val ue	Effect SizeCramer V
Age at first use of cigarettes or nicotine						
Never used	10 (13.7)	13 (13.8)	2094 (62.4)	LR = 215.254; df = 6	<0.001 ^*^	0.197
Less than 14 years	15 (21.9)	28 (29.8)	156 (4.6)			
15 to 17 years	34 (46.6)	34 (36.2)	479 (14.3)			
18 years or older	13 (17.8)	19 (20.2)	628 (18.7)			
Frequency of e-cigarette use						
Never	15 (23.8)	28 (34.6)	733 (58.1)	LR = 73.390; df = 8	<0.001 ^*^	0.195
Not within past year	12 (19.0)	29 (35.8)	260 (20.6)			
Rarely	19 (30.2)	13 (16.0)	199 (15.8)			
Occasionally	6 (9.5)	9 (11.1)	46 (3.6)			
Daily	11 (17.5)	2 (2.5)	23 (1.8)			
Frequency of alcohol consumption						
Never	4 (5.5)	10 (10.6)	649 (19.3)	LR = 64.542; df = 8	<0.001 ^*^	0.105
Monthly or less	7 (9.6)	10 (10.6)	654 (19.5)			
2 to 4 times a month	16 (21.9)	28 (29.8)	1092 (32.5)			
2 to 3 times a week	27 (37.0)	35 (37.2)	740 (22.1)			
4+ times a week	19 (26.0)	11 (11.9)	221 (6.6)			
AUDIT score ≥8 (%)	51 (69.9)	48 (51.1)	768 (22.9)	LR = 101.643; df = 2	<0.001 ^*^	0.186
DAST-10 score ≥3 (%)	48 (65.8)	40 (42.6)	201 (6.0)	LR = 255.421; df = 2	<0.001 ^*^	0.373
Nonprescription amphetamines						
Never	57 (78.1)	65 (69.1)	1206 (97.2)	LR = 126.089; df = 8	<0.001 ^*^	0.297
In past, not within past 12 months	4 (5.5)	24 (25.5)	18 (1.5)			
Rarely	7 (9.6)	2 (2.1)	13 (1.0)			
Occasionally	3 (4.1)	3 (3.2)	1 (0.1)			
Daily	2 (2.7)	0 (0.0)	3 (0.2)			
Cocaine						
Never	36 (49.3)	29 (31.2)	1059 (86.2)	LR = 178.490; df = 6	<0.001 ^*^	0.295
In past, not within past 12 months	16 (21.9)	45 (48.4)	99 (8.1)			
Rarely	15 (20.5)	16 (17.2)	58 (4.7)			
Occasionally	6 (8.2)	3 (3.2)	12 (1.0)			
Daily	0 (0.0)	0 (0.0)	0 (0.0)			
Opiates						
Never	57 (78.1)	75 (80.6)	1223 (98.6)	LR = 103.662; df = 8	<0.001 ^*^	0.272
In past, not within past 12 months	8 (11.0)	17 (18.3)	12 (1.0)			
Rarely	4 (5.5)	1 (1.1)	1 (0.1)			
Occasionally	1 (1.4)	0 (0.0)	2 (0.2)			
Daily	3 (4.1)	0 (0.0)	2 (0.2)			
Inhalants						
Never	58 (79.5)	80 (86.0)	1210 (98.1)	LR = 74.952; df = 6	<0.001 ^*^	0.236
In past, not within past 12 months	8 (11.0)	13 (14.0)	18 (1.5)			
Rarely	7 (9.6)	0 (0.0)	3 (0.2)			
Occasionally	0 (0.0)	0 (0.0)	3 (0.2)			
Daily	0 (0.0)	0 (0.0)	0 (0.0)			
Hallucinogens						
Never	23 (31.5)	26 (27.7)	964 (77.9)	LR = 178.086; df = 8	<0.001 ^*^	0.293
In past, not within past 12 months	17 (23.3)	47 (50.0)	161 (13.0)			
Rarely	21 (28.8)	18 (19.1)	76 (6.1)			
Occasionally	10 (13.7)	3 (3.2)	37 (3.0)			
Daily	2 (2.7)	0 (0.0)	0 (0.0)			
Marijuana						
Never	3 (4.1)	0 (0.0)	42 (3.4)	LR = 85.914; df = 8	<0.001 ^*^	0.186
In past, not within past 12 months	16 (21.9)	37 (39.4)	328 (26.4)			
Rarely	5 (6.8)	21 (22.3)	444 (35.7)			
Occasionally	24 (32.9)	19 (20.2)	341 (27.5)			
Daily	25 (34.2)	17 (18.1)	87 (7.0)			
Prescription pain medication						
Never	25 (34.2)	22 (23.7)	1097 (88.5)	LR = 333.028; df = 8	<0.001 ^*^	0.457
In past, not within past 12 months	13 (17.9)	63 (67.7)	110 (8.9)			
Rarely	23 (31.5)	7 (7.5)	29 (2.3)			
Occasionally	9 (12.3)	0 (0.0)	3 (0.2)			
Daily	3 (4.1)	1 (1.1)	1 (0.1)			

Data refer to N (percentage).

^*^P < 0.05, Bonferroni-corrected.

Table 3 presents the sexual behavior of participants. Sedative/tranquilizer misuse was significantly associated with being sexually active at a younger age and engaging sex without barrier contraception.

Mental health histories are presented in Table 4. Sedative/tranquilizer misuse was significantly associated with higher rates of depression, PTSD (almost one-half screening positive for PTSD), ADHD (approximately 40% screening positive for ADHD), and anxiety. In addition, those who misused sedative/tranquilizers were more likely to report poorer self-esteem. Sedative/tranquilizer misuse was neither significantly associated with gambling disorder or binge-eating disorder, nor with significantly higher caffeine use.

**TABLE 4:** Impulsive Behaviors and Psychiatric History of University Students Based on Nonmedical Use of Prescription Sedative/Tranquilizers

Variable	Students Who Currently Misuse Prescription Sedative/Tranquilizers (n = 73)	Students Who Have Misused Prescription Sedative/Tranquilizers in the Past (n = 94)	Students Who Have Never Misused Prescription Sedative/Tranquilizers (n = 3358)	StatisticLikelihood Ratio	*P* Value	Effect Size Cramer V
Amount of caffeinated soft drinks consumed over the past week, n (%)						
Never	34 (47.2)	40 (43.5)	1590 (48.2)	LR = 22.345; df = 10	0.011	0.070
1 to 2 drinks	13 (18.1)	30 (32.6)	1076 (32.6)			
3 to 6 drinks	12 (16.7)	12 (13.0)	420 (12.7)			
7 to 12 drinks	5 (6.9)	6 (6.5)	145 (4.3)			
13 to 23 drinks	6 (8.3)	3 (3.3)	47 (1.4)			
24 or more drinks	2 (2.8)	1 (1.1)	24 (0.7)			
Gambling disorder?	3 (20.0)	1 (6.7)	10 (3.7)	LR = 5.207; df = 2	0.074	0.169
Positive screen						
Binge eating disorder?	4 (5.6)	5 (5.8)	74 (2.3)	LR = 5.208	0.074	0.045
Positive screen						
Has been treated for drug/alcohol use problems	7 (9.7)	14 (15.2)	41 (1.2)	LR = 55.832; df = 2	<0.001 ^*^	0.190
Yes						
Has been treated for psychological/emotional problems						
Yes	43 (59.7)	53 (57.6)	930 (28.4)	LR = 61.364; df = 2	<0.001 ^*^	0.141
Currently taking prescribed mental health mediation (s)	34 (47.2)	32 (34.8)	408 (12.4)	LR = 77.313; df = 2	<0.001 ^*^	0.176
Yes						
Has used drugs in order to lose weight	19 (39.6)	13 (23.6)	103 (6.8)	LR = 51.885; df = 2	<0.001 ^*^	0.226
Yes						
PHQ-9 Total
Score of 10 or more	13 (18.1)	6 (6.8)	134 (4.2)	LR = 19.837; df = 2	<0.001 ^*^	0.098
PTSD						
Positive screen	33 (46.5)	23 (25.3)	434 (13.4)	LR = 51.849; df = 2	<0.001 ^*^	0.144
Anxiety total						
Grouped						
No Anxiety (score 0–4)	18 (25.0)	34 (37.8)	1897 (59.5)	LR = 67.314; df = 6	<0.001 ^*^	0.114
Mild (score 5–9)	21 (29.2)	36 (40.0)	751 (23.5)			
Moderate (score 10–14)	13 (18.1)	11 (12.2)	348 (10.9)			
Severe (score 15–21)	20 (27.8)	9 (10.0)	193 (8.1)			
ADHD	28 (39.4)	27 (29.7)	535 (16.7)	LR = 28.440; df = 2	<0.001 ^*^	0.101
Positive screen						
Rosenberg Self-Esteem Scale	22 (31.0)	19 (21.1)	450 (14.3)	LR = 15.173; df = 2	0.001 ^*^	0.074
Less than 15						

Data refer to N (percentage).

^*^P < 0.05, Bonferroni-corrected. PTSD, post-traumatic stress disorder.

In terms of psychological traits (see Table 5), those who misused sedative/tranquilizers reported significantly greater scores of impulsivity on all subscales of the BIS-11, and greater levels of compulsive traits on the CHI-T.

**TABLE 3:** Sexual Behavior in University Students Based on Nonmedical Use of Prescription Sedative/Tranquilizers

Variable	Students Who Currently Misuse Prescription Sedative/Tranquilizer (n = 73)	Students Who Have Misused Prescription Sedative/Tranquilizer in the Past (n = 94)	Students Who Have Never Misused prescription Sedative/Tranquilizer (n = 3358)	StatisticLikelihood Ratio	*P* Value	Effect Size Cramer V
Has been sexually active						
Yes	67 (91.8)	88 (94.6)	2394 (72.0)	LR = 47.678; df = 2	<0.001 ^*^	0.103
No	6 (8.2)	5 (5.4)	932 (28.0)			
Age at first sexual activity with another						
<11 years	1 (1.5)	2 (2.3)	18 (0.8)	LR = 59.563; df = 8	<0.001 ^*^	0.114
12 to 14 years	11 (16.4)	16 (18.2)	129 (5.4)			
15 to 17 years	38 (56.7)	47 (53.4)	977 (40.9)			
18 to 20 years	14 (20.9)	20 (22.7)	952 (39.8)			
21 years or older	3 (4.5)	3 (3.4)	313 (13.1)			
Frequency of physical barrier use						
<50% of the time	28 (41.8)	45 (51.1)	909 (38.1)	LR = 23.809; df = 6	0.001 ^*^	0.067
50 to 75% of the time	9 (13.4)	9 (10.2)	215 (9.0)			
76 to 95% of the time	18 (26.9)	16 (18.2)	384 (16.1)			
96 to 100% of the time	12 (17.9)	18 (20.5)	878 (36.8)			

Data refer to N (percentage).

^*^P < 0.05 Bonferroni-corrected.

**TABLE 5:** Impulsivity and Compulsivity of University Students Based on Nonmedical Use of Prescription Sedative/Tranquilizers

Variable	Students Who Currently Misuse Prescription Sedative/Tranquilizers (n = 73)	Students Who have Misused Prescription Sedative/Tranquilizers in the Past (n = 94)	Students Who Have Never Misused Prescription Sedative/Tranquilizers (n = 3358)	StatisticANOVA	*P* Value	Effect SizeCohen d
Cambridge-Chicago Compulsivity Trait Scale Mean (SD)	15.78 (16.27)^†,‡^	10.91 (14.34)^‡^	9.14 (13.41)^†^	F (2,3412) = 8.596	<0.001 ^*^	0.2831
Barratt Impulsiveness Scale (BIS-11) Total Score Mean (SD)	66.17 (10.37) ^†^	65.08 (11.73)^‡^	59.12 (10.03)^†,‡^	F (2,3184) = 30.309	<0.001 ^*^	0.6388
Attentional impulsiveness Mean (SD)	19.14 (4.17) ^†^	18.40 (4.48)^‡^	16.08 (3.92)^†,‡^	F (2,3227) = 35.116	<0.001 ^*^	0.6725
Non-planning impulsiveness Mean (SD)	25.17 (4.56) ^†^	24.13 (5.24)^‡^	22.85 (4.73)^†,‡^	F (2,3271) = 10.966	<0.001 ^*^	0.3677
Motor impulsiveness Mean (SD)	22.31 (4.67)^†^	22.39 (4.29)^‡^	20.22 (3.89)^†,‡^	F (2,3285) = 22.536	<0.001 ^*^	0.5441

Superscript symbols (^†;‡^) indicate post hoc Bonferroni test for significance: the mean difference is significant at the 0.05 level. Data refer to mean and (standard deviation [SD]).

^*^P < 0.05 Bonferroni-corrected.

## DISCUSSION

This study examined the prevalence of the misuse of sedative/tranquilizers in a large sample of university students; and ways in which sedative/tranquilizer misuse was related to concomitant use of other drugs as well as mental health and psychosocial functioning. Misuse in this context was defined as intake of these substances by individuals who had not been prescribed them. We found that 2.1% of the sample reported past 12-month misuse of sedative/tranquilizers (with 2.8% having ever misused them). Overall, the lifetime rates found in our study (almost 5%) are similar to those reported in the National Survey on Drug Use and Health, where 1.6% misused tranquilizer medication and 0.2% misused sedative medication (SAMHSA, 2017); and to those reported in the NESARC study, which found a lifetime rate of 5.5% of misuse of prescription tranquilizer or sedative medications (Boyd et al., 2018). Overall, sedative/tranquilizer misuse appears to be particularly high in young adults, and these findings are concerning regarding the long-term effects of this misuse during young adulthood. Although the NESARC longitudinal data found that 79% of adults who engaged in tranquilizer or sedative misuse at the initial assessment had stopped using these drugs at follow-up 3 years later, 45% met criteria for at least 1 other substance use disorder at the follow-up assessment, particularly in those who were aged 18 to 25 years at initial assessment (Boyd et al., 2018). What is not answerable at this time, however, is how to predict on the individual level whether a young adult who misuses sedative/tranquilizers will progress to another substance use disorder in later life. The relatively high rates of concurrent use of other substances (such as opiates) we found in those who misuse sedatives/tranquilizers is troubling because such different types of substances can have dangerous acute synergistic effects (Jones et al., 2012).

The current study found that university students who reported misuse of sedative/tranquilizers (ever use/past year use) had significantly higher rates of several types of substance use and higher impulsivity and compulsivity. In particular, from an impulsivity perspective, they exhibited greater likelihood of being sexually active, earlier age at first sexual activity with another, and less use of barrier contraception. The potential impact of risky sexual practices can be profound for an individual, such as putting oneself into vulnerable situations or being exposed to higher risk of sexually transmitted diseases. Additionally, sedative/tranquilizer use was associated with elevated ADHD symptoms, and trait impulsiveness on the BIS, which again may be in keeping with a propensity towards risk in those who misuse sedatives/tranquilizers. We also assessed trait compulsivity using a recently developed scale, finding that sedative/tranquilizer use was associated with elevated scores.

Collectively, these findings suggest a more prominent general risk-taking, that is, impulsive profile in university students who misuse sedative/tranquilizers. We also found evidence of heightened compulsivity, which is interesting because of a hypothesized shift from impulsive to compulsive substance use over time, primarily observed to date in preclinical models (Belin et al., 2008; Dalley et al., 2011). Here, it could be reasoned that the study participants would be—on average—at relatively early stages of addiction (ie, impulsive rather than compulsive) because of their young age.

There are, however, several potential explanations for these impulsive/compulsive associations with sedative/tranquilizer use, which are not mutually exclusive. Sedative/tranquilizer use could lead to other drug use, such as by causing disinhibition (Deakin et al., 2004) or reducing fear to use other drugs. Alternatively, use of other drugs (eg, stimulants) could lead to anxiety, which in turn individuals seek to ameliorate by using sedatives/tranquilizers. Furthermore, trait impulsivity may predispose towards these multiple manifest types of behavior separately via a common latent mechanism (ie, via latent phenotypes; Chamberlain et al., 2018b, 2019).

Another, related explanation that could potentially drive the misuse of sedatives/tranquilizers in university students might relate to the other mental health problems reported by these students. In this study, the misuse of sedatives/tranquilizers was significantly associated with symptoms of PTSD, anxiety, depression, and ADHD. The conventional explanation for these associations would be that people self-medicate with substances to address their mental health problems. Although that is possible on an individual level, this theory of self-medication may be either too simplistic or even incorrect for many people (Chambers, 2010). In fact, research suggests that the co-occurrence of multiple drugs used in problematic patterns may be linked to their shared vulnerability with other types of mental illness (Sentir et al., 2018). The high rates of PTSD in our sample could theoretically have occurred if students included many people from a military background (military veterans); however, this was not assessed.

To our knowledge, this is the first study to examine associations among sedative/tranquilizer misuse, demographic variables, academic performance, mental health problems and substance misuse, and measures of impulsivity and compulsivity in a large university sample. Furthermore, an anonymous survey may have increased openness of study participants to report underlying mental health problems and substance use. Nonetheless, several limitations should be noted. First, as a cross-sectional study, the direction of causality cannot be determined. For example, this study design cannot assess the extent to which impulsive traits predispose to sedative and other drug use, as compared with sedative drugs having direct effects on mental health. Second, the use of online surveys has inherent limitations, such as diagnostic accuracy and veracity. Third, findings were generally of small or medium effect size; nonetheless, even small effects may be important from a public health point of view. Fourth, the self-selected nature of participation may have resulted in a lack of representativeness of the background population. Fifth, sedative/tranquilizers misuse was separated into 3 broad categories: never, lifetime but not the past 12 months, and the past 12 months use. Furthermore, we defined misuse as use of these substances in the absence of legitimate prescription to the person, rather than misuse of medications legitimately prescribed to the person. As such, our definition would capture many but not all cases of misuse as defined by the World Health Organization (“Use of a substance for a purpose not consistent with legal or medical guidelines, as in the non-medical use of prescription medications.”) (WHO, 2006). More detailed inquiry of frequency, nature, and pattern of sedative/tranquilizer misuse (eg, lifetime could mean a single use or daily in the past) and specific sedative/tranquilizer used would provide a more in-depth analysis of college students’ sedative/tranquilizer misuse and psychosocial functioning. Lastly, we wished to present the group differences in actuality (ie, as people present in reality), rather than using covariates. Use of multinomial or other regression models to control for covariates would be unlikely to be valid in this setting, as many measures are expected to be related to each other statistically (eg, different drugs being used, impulsivity, etc) and statistical assumptions of noncollinearity are likely to be breached.

In conclusion, we found that sedative/tranquilizer misuse in young adults was associated with lower grade point averages, elevated occurrence of multiple mental disorders (including ADHD, depression, anxiety, and PTSD), and riskier sexual practices (earlier age of first sexual activity and less use of barrier contraception). Furthermore, sedative/tranquillizer misuse was associated with significantly elevated trait impulsivity and compulsivity. Longitudinal research is needed to better understand whether these associations are causal, particularly, whether latent phenotypes of impulsivity and compulsivity might in fact predispose towards a range of problematic behaviors in young people.
